# Challenges in genetic counseling for congenital anomalies of the kidneys and urinary tract (CAKUT) spectrum

**DOI:** 10.1515/crpm-2021-0063

**Published:** 2022-04-20

**Authors:** Ping Gong, Myriam Pelletier, Neil Silverman, Kathleen Kuhlman, Robert Wallerstein

**Affiliations:** Integrated Genetics, Genetic Counseling and Services, Laboratory Corporation of America, Monrovia, CA, USA; Department of Obstetrics and Gynecology, David Geffen School of Medicine at UCLA, Center for Fetal Medicine and Women’s Ultrasound, Los Angeles, CA, USA; Valley Perinatal Services, Maternal Fetal Medicine, Phoenix, AZ, USA

**Keywords:** CAKUT, congenital anomalies of the kidneys and urinary tract, genetic counseling, genetic evaluation, genetic testing, molecular genetics, prenatal care, prenatal diagnosis, recurrent renal agenesis, renal agenesis

## Abstract

**Objectives:**

Congenital anomalies of the kidneys and urinary tract (CAKUT) are one of the most common sets of congenital defects. Bilateral renal agenesis is a severe presentation of the CAKUT spectrum.

**Case presentation:**

We report on two families who presented with recurrent pregnancies affected with bilateral renal agenesis and negative family histories. Likely pathogenic variants in the *GREB1L* gene were identified in the affected pregnancies and subsequently in their asymptomatic fathers. The first familial variant was identified by a multi-gene CAKUT panel and the second by whole exome sequencing. Renal ultrasound showed the father in family 1 had asymptomatic unilateral pelvic kidney and the father in family 2 had no apparent renal anomalies.

**Conclusions:**

Recent identification of genes responsible for CAKUT allows for genetic testing of affected families. Identification of the genetic etiology of CAKUT cases has multiple benefits including accurate risk assessment and reproductive options. Genetic counseling around CAKUT is challenging due to the extreme variability in presentation of the disorders.

## Introduction

Congenital anomalies of the kidney and urinary tract (CAKUT) include a wide spectrum of structural malformations resulting from defects in the morphogenesis of the kidney and of the urinary tract [1]. CAKUT range from complete renal agenesis to renal hypodysplasia, multicystic kidney dysplasia, duplex renal collecting system, ureteropelvic junction obstruction, horseshoe kidney, hydronephrosis, megaureter, and posterior urethral valve [1, 2]. The most commonly used classification is based on anatomical characteristics (the segment of the urinary tract involved), with ureteropelvic junction obstruction being the most frequent observed phenotype (20%). Altogether, CAKUT are one of the most common sets of congenital defects, affecting 3–7 out of 1,000 live births and represent more than 20% of birth defects. CAKUT are responsible for 40–50% of pediatric and 7% of adult end-stage renal disease worldwide [1]. It is estimated that bilateral renal agenesis occurs at a frequency of 1/3,000–1/5,000, while unilateral renal agenesis occurs more frequently in up to 1/1,000 births [3]. CAKUT are commonly detected with prenatal sonography; however, many cases remain undiagnosed until adulthood and represent a large spectrum of diseases with highly variable grades of severity and outcome. Moreover, CAKUT can appear as an isolated feature or as part of a syndromic condition with extra-renal manifestations, making diagnosis and clinical classification even more challenging [1]. The etiology of the CAKUT spectrum is complex and multifactorial involving genetic and non-genetic factors [1, 4]. In recent years, alterations in more than 75 genes have been shown to cause isolated or syndromic CAKUT, in an autosomal dominant or, less frequently, recessive mode of inheritance [5]. In addition, copy number variations have been widely associated with CAKUT spectrum [6, 7]. Despite recent advances, genetic diagnosis of CAKUT is still challenging due to their genetic and phenotypic heterogeneity as well as incomplete penetrance. Consensus guidelines on genetic evaluation of CAKUT is currently lacking.

*GREB1L* was identified as a CAKUT-susceptibility gene in 2017 [3, 5, 8]*.* It has been reported as a target of retinoic acid signaling, a pathway that is crucial for renal development. Dysregulation of this pathway has been proposed as a causative mechanism for urinary tract malformations [8]. Mutations in *GREB1L* have been associated with a form of renal hypodysplasia/aplasia (RHDA3) (OMIM#617805), which is inherited in an autosomal dominant pattern and presents as early as fetal life. The phenotype is characterized by high variability within and among families, as well as incomplete penetrance. Bilateral renal agenesis, as seen in our families, is typically associated with perinatal demise, either prenatally due to complications of severe oligohydramnios, or postnatally due to pulmonary and metabolic issues related to absence of normal renal function. Individuals with this genotype, however, may instead have unilateral agenesis that is compatible with life, or milder manifestations, such as vesicoureteral reflux (VUR). Female mutation carriers may also have uterine or ovarian abnormalities ranging from uterine agenesis with bilateral renal agenesis to milder defects such as unicornuated uterus and unilateral ovarian and fallopian tube agenesis [8]. In a study of 183 unrelated families affected by various forms of CAKUT, 16 (8.7%) heterozygous pathogenic or suspected pathogenic variants in *GREB1L* were identified, 12 of which were found in 54 cases (25.8%) with bilateral renal agenesis in this cohort [8]. In another study of 612 individuals affected by renal agenesis and hypodysplasia, 17 (2.8%) heterozygous pathogenic or suspected pathogenic variants in *GREB1L* were identified [5].

## Case presentation

### History of family 1

**Figure 1:**
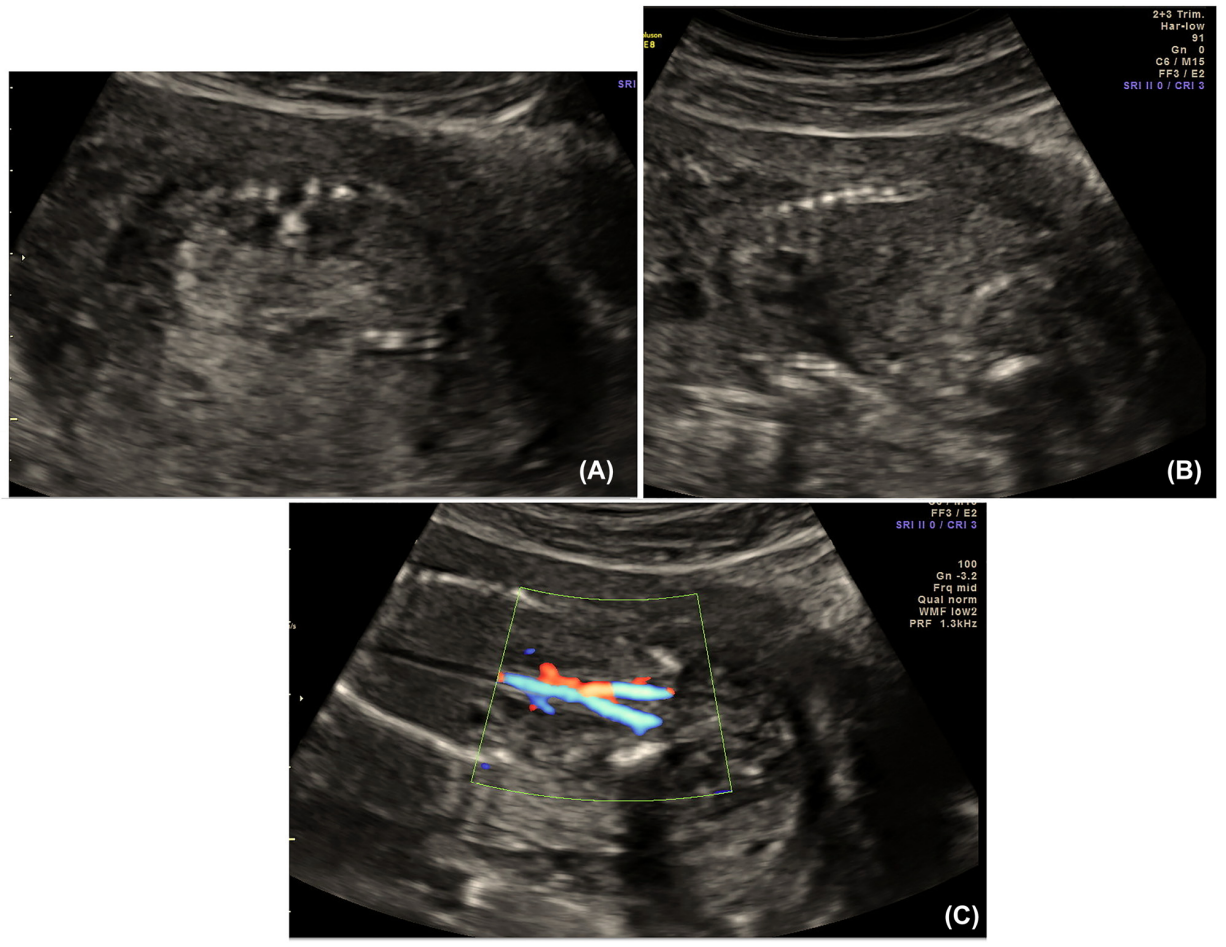
Ultrasound images of proband of family 1. (A) Anhydramnios and absent renal fossae. (B) Anhydramnios. (C) Absence of renal artery blood flow from aorta.

**Figure 2:**
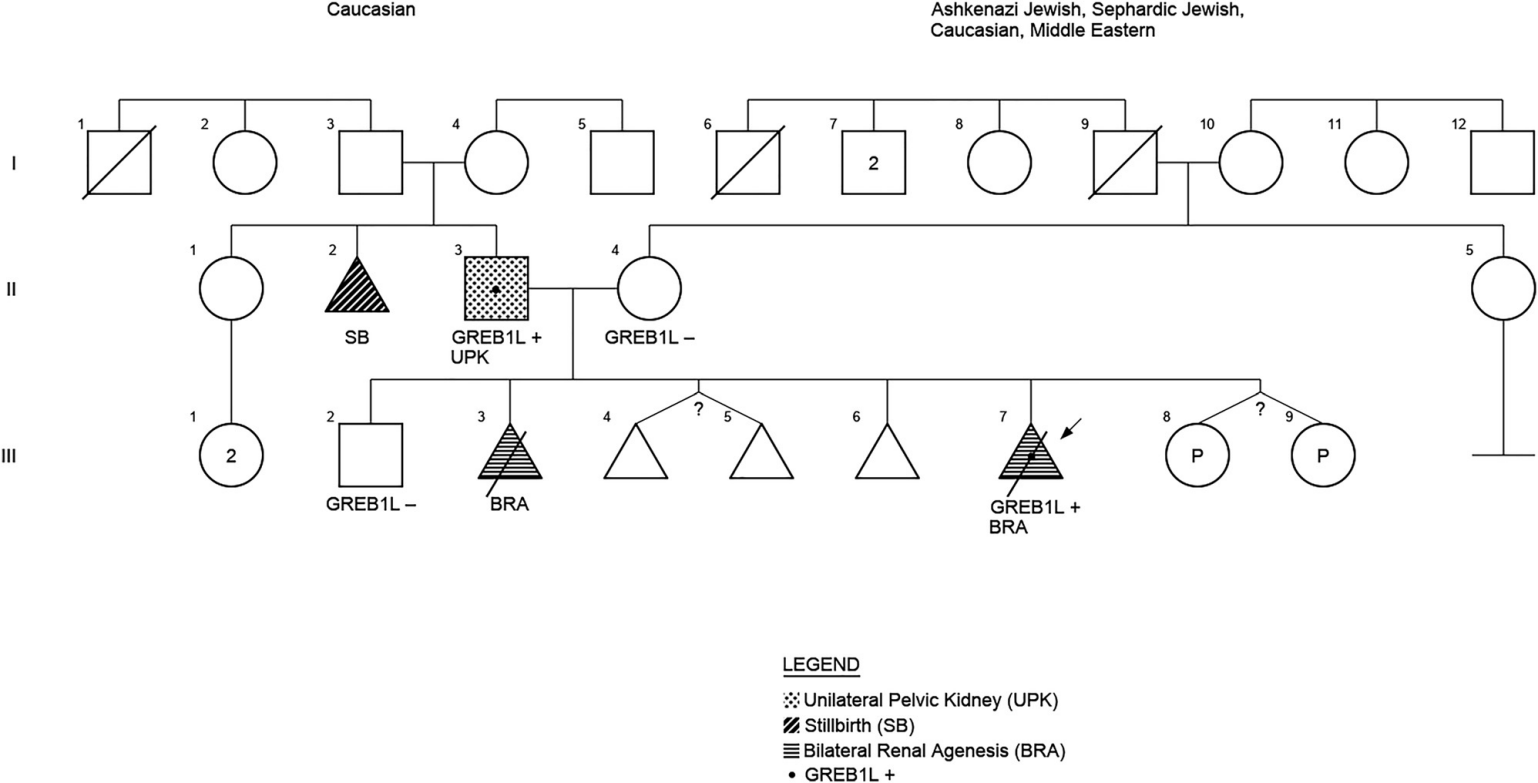
Pedigree of family 1.

Family 1 first presented to genetic counseling with a pregnancy found to be affected with fetal bilateral renal agenesis at 16 weeks gestation (Figure 1). The fetal bladder was not visualized though branched umbilical arteries were seen on color flow in the region of the bladder. Echogenic fetal bowel was seen. Inquiry of the couple’s reproductive history revealed a previous pregnancy also affected with fetal bilateral renal agenesis, in addition to clubfeet, single umbilical artery, non-visualized fetal bladder and anhydramnios; these anomalies were identified at 20 weeks 5 days gestation. The previous pregnancy was electively terminated at 21 weeks gestation due to poor prognosis; no kidney tissue was identified on gross examination of the products of conception. Chromosomal microarray of the products of conception revealed a normal female genotype. In addition, the couple’s reproductive history included a healthy male offspring, spontaneous abortion of a twin pregnancy at 8 weeks gestation due to an unknown etiology, as well as a chemical pregnancy. The couple both denied any personal and family histories of CAKUT, although the father did report that one of his siblings was stillborn due to an unknown etiology (Figure 2).

The pregnancy was terminated at 17 weeks gestation due to poor prognosis. Based on its recurrence, the bilateral renal agenesis was suspected to have a genetic etiology. A CAKUT multi-gene panel was performed on the products of conception of this second affected pregnancy. A likely pathogenic variant, c.5356_5357del (p.Gln1786Valfs*13), was identified in *GREB1L*. This sequence variant was predicted to result in a frameshift and premature protein termination. This variant had not been reported previously but was expected to be pathogenic. Fetal chromosomal microarray revealed a normal female genetic complement. Targeted analysis of the *GREB1L* variant was subsequently performed on the parents; the father was found to carry the variant, while the mother was negative. Although the father previously denied any personal history of CAKUT, his abdominal/pelvic ultrasound revealed a unilateral pelvic kidney. The mother’s renal ultrasound was normal. The couple’s son had targeted analysis of the likely pathogenic variant in *GREB1L* and was negative. Although he had not had postnatal renal evaluation, his prenatal ultrasound had been unremarkable.

At the time of writing this manuscript, the patient was nearing the end of a new pregnancy, achieved via conception after ovulation induction, resulting in diamniotic-dichorionic female twins. The couple was offered both 1st trimester and 2nd trimester prenatal diagnostic testing for the *GREB1L* mutation but declined, since they had decided they would not consider pregnancy termination or selective reduction based solely on the fetuses’ genetic statuses, given the variable expression seen within their own family. An early fetal structural ultrasound at 16 weeks gestation demonstrated no evidence of genitourinary anomalies in either twin, and subsequent ultrasounds into the third trimester demonstrated normal anatomy, including the urinary systems, in both fetuses (Figure 2).

### History of family 2

**Figure 3:**
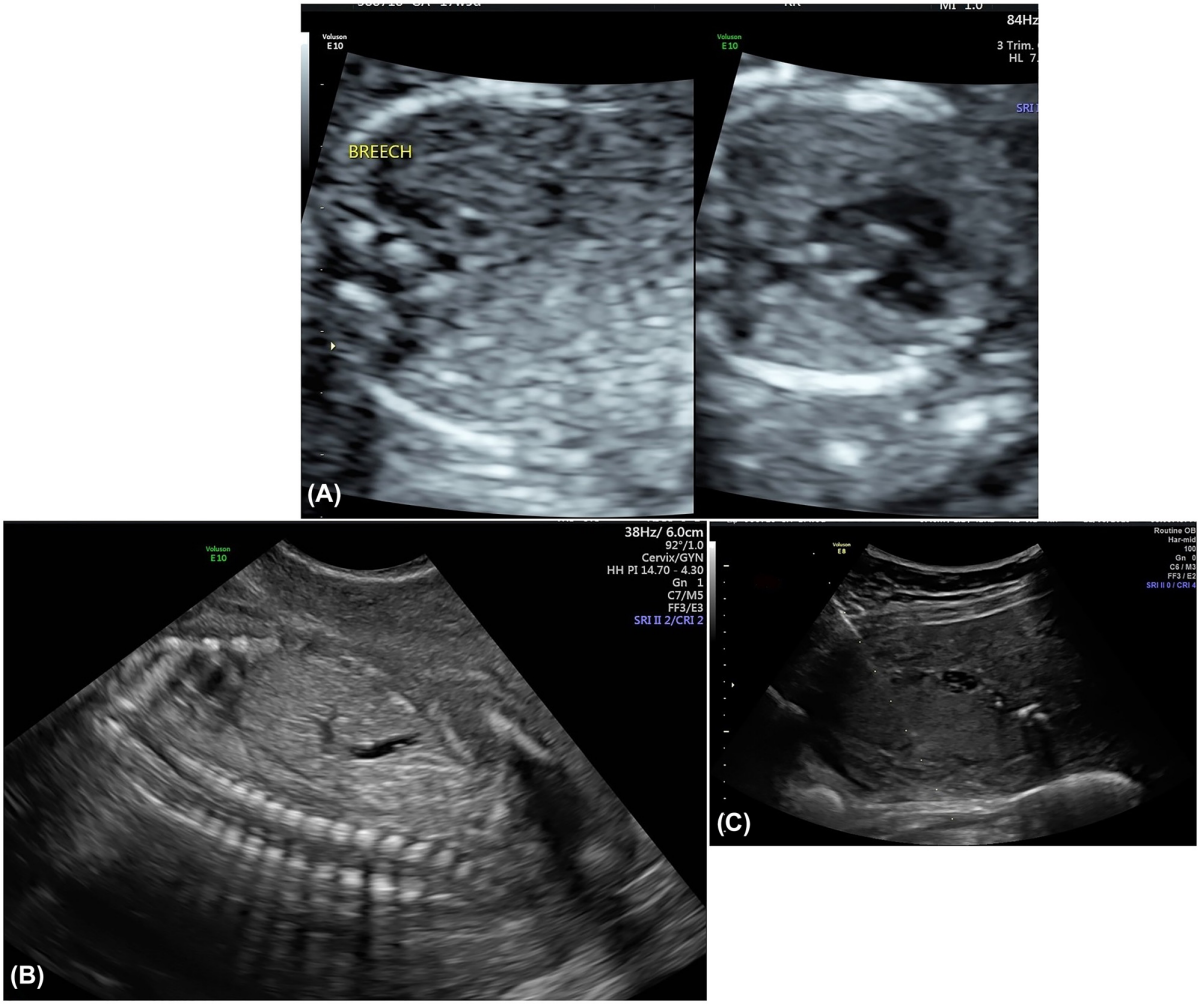
Ultrasound images of proband of family 2. (A) Transverse views of fetus that show empty renal fossa bilaterally and normally oriented heart. (B) Anhydramnios and absence of fluid filled stomach and bladder. (C) Placental biopsy.

**Figure 4:**
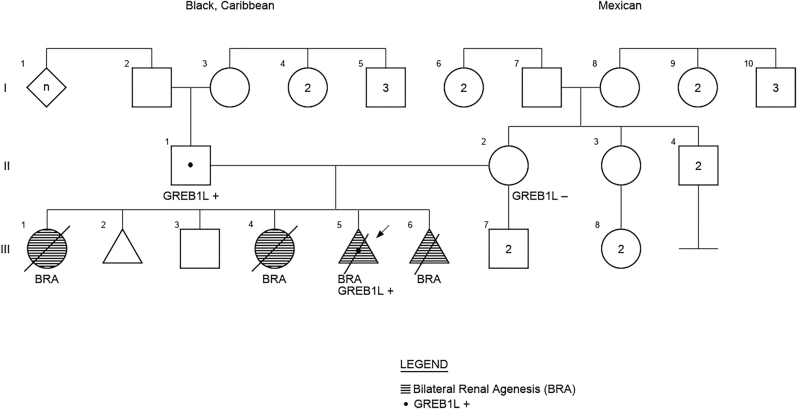
Pedigree of family 2.

Family 2 was referred to genetic counseling due to suspected recurrence of bilateral renal agenesis. The couple had two previous female pregnancies affected with bilateral renal agenesis that resulted in preterm neonatal death. The couple’s reproductive history also included an unaffected son (not tested) and one early miscarriage. For the current pregnancy, a detailed fetal anatomic survey performed at 17 weeks 5 days showed a smaller sized fetus measuring 16 weeks 1 day, right renal agenesis, a very small and suspected nonfunctional left kidney, absent stomach, absent bladder, small thorax, an echogenic intracardiac focus, and anhydramnios (Figure 3). Placental biopsy was performed for genetic studies prior to termination of pregnancy. Results of chromosome microarray analysis showed a normal female complement. Whole exome sequencing trio results showed a variant in the *GREB1L* gene, c.5622T>A (p.Cys1874X), which was classified as likely pathogenic. This variant had not been previously reported but was predicted to introduce a premature termination codon in the GREB1L protein, which was predicted to result in truncation or loss of the GREB1L protein via nonsense mediated decay. The variant was also detected in the father. The couple denied any personal or family history of CAKUT (Figure 4). The couple also reportedly had normal renal ultrasounds.

At the time of writing this manuscript, the couple had experienced a subsequent pregnancy of a male fetus with anhydramnios, and suspected bilateral renal agenesis based on absence of the fetal bladder and inability to see fetal kidneys and renal arteries at 19 weeks 6 days gestation. The couple elected to undergo termination of this pregnancy due to poor prognosis. Prenatal diagnosis was declined because the option of placental biopsy was unavailable locally.

## Discussion

The identification of CAKUT genes is changing the practice of prenatal diagnosis for families with a history of renal anomalies. Prenatal chromosomal microarray analysis has been a standard recommendation for evaluation of all major fetal structural abnormalities including renal agenesis (ACOG 682). Our experience suggests that when renal agenesis is identified, testing for CAKUT genes should also be considered especially when chromosomal microarray results are non-diagnostic. A number of studies in the mid-2010s demonstrated that targeted panels had a diagnostic yield of 6–10% in patients with isolated CAKUT [4, 9]. The diagnostic yield is likely higher as mutation panels are updated when new genes are identified. In addition, as the list of defined syndromes grows and the cost of sequencing declines, whole exome sequencing or genome sequencing will likely emerge as the preferred primary diagnostic modality [7]. In recent studies, the diagnostic yield of whole exome sequencing has been reported to be as high as 14.5% in isolated CAKUT cases, and higher in syndromic cases [10], [11], [12]. When renal agenesis is diagnosed in the second and third trimester and oligohydramnios does not allow for amniocentesis, prenatal diagnosis via placental biopsy can allow for a genetic diagnosis. In cases where prenatal diagnosis is not possible due to technical factors or parental choice, genetic testing after delivery is appropriate.

The CAKUT in both of the families appeared to be autosomal recessive on initial clinical evaluation, with affected female probands who are sibs and reportedly negative family history. However, genetic testing proved the conditions to be autosomal dominant in both families. In family 1, an asymptomatic CAKUT (unilateral pelvic kidney) was discovered in the mutation-carrier father on subsequent renal evaluation; this exemplified the condition’s variable expressivity. In family 2, the carrier father had normal imaging studies of the renal and urinary system, which demonstrated the condition’s reduced penetrance.

Based on our experience, due to CAKUT’s incomplete penetrance and intra-familial variable expressivity, apparently sporadic cases may in fact be familial, with additional affected family members yet to be diagnosed. Autosomal dominant inheritance should be considered even when the known affected individuals are within the same sibship and inheritance may appear to be autosomal recessive. For the same reasons, clinical evaluation of the genitourinary system should be considered in apparently asymptomatic first degree relatives, including parents and siblings. If a clinically significant variant for an autosomal dominant condition is identified in the proband, testing first degree relatives for the variant should be considered to establish inheritance pattern in the family. In addition, genetic testing of unaffected family members, including parents and siblings, is appropriate to help establish disease penetrance in the family and allow for more accurate genetic counseling to other at risk individuals in the future.

In the majority of reported cases where *GREB1L* variants were identified in patients with bilateral renal agenesis, the mildly affected or unaffected mother is the carrier. This observation led to the hypotheses that the *GREB1L* gene is imprinted, with specific expression of the maternal allele during kidney development (paternally imprinted gene) or that *GREB1L* variants would affect male fertility, explaining the low rate of paternal inheritance [8]. Our cases do not support these hypotheses.

Identification of the genetic etiology of renal agenesis helps define recurrence risks, allows for early prenatal diagnosis and preimplantation genetic testing (PGT), and informs subsequent diagnosis and follow up of family members and pregnancies that are at risk for CAKUT. The anomalies seen in CAKUT spectrum are greatly variable, and as such, the time for detection by ultrasound is variable as well. Serial ultrasonography in at risk pregnancies (e.g., pregnancies with a positive genotype and at-risk pregnancies where prenatal diagnosis was declined) would be advised, as CAKUT findings may not be detectable until later in pregnancy or post-natal life. Although the aforementioned genetic and clinical evaluations provide many benefits, challenges remain in genetic counseling for CAKUT especially in the prenatal setting. The variable expressivity and reduced penetrance pose significant difficulties for clinicians to provide prognostic information on mutation-positive fetuses that have yet to demonstrate clinical signs of the condition. The extremely broad prognosis makes it difficult for prospective parents to make decisions regarding continuation or termination of a genetically affected pregnancy. Identification of additional genetic and environmental modifiers of the CAKUT phenotype can potentially help with these remaining challenges.

## References

[1] Capone VP, Morello W, Taroni F, Montini G (2017). Genetics of congenital anomalies of the kidney and urinary tract: the current state of play. Int J Mol Sci.

[2] Rodriguez MM (2014). Congenital anomalies of the kidney and the urinary tract (CAKUT). Fetal Pediatr Pathol.

[3] Brophy PD, Rasmussen M, Parida M, Bonde G, Darbro BW, Hong X (2017). A gene implicated in activation of retinoic acid receptor targets is a novel renal agenesis gene in humans. Genetics.

[4] Nicolaou N, Renkema KY, Bongers EM, Giles RH, Knoers NV (2015). Genetic, environmental, and epigenetic factors involved in CAKUT. Nat Rev Nephrol.

[5] Sanna-Cherchi S, Khan K, Westland R, Krithivasan P, Fievet L, Rasouly HM (2017). Exome-wide association study identifies GREB1L mutations in congenital kidney malformations. Am J Hum Genet.

[6] Verbitsky M, Westland R, Perez A, Kiryluk K, Liu Q, Krithivasan P (2019). The copy number variation landscape of congenital anomalies of the kidney and urinary tract. Nat Genet.

[7] Sanna-Cherchi S, Westland R, Ghiggeri GM, Gharavi AG (2018). Genetic basis of human congenital anomalies of the kidney and urinary tract. J Clin Invest.

[8] De Tomasi L, David P, Humbert C, Silbermann F, Arrondel C, Tores F (2017). Mutations in GREB1L cause bilateral kidney agenesis in humans and mice. Am J Hum Genet.

[9] Hwang DY, Dworschak GC, Kohl S, Saisawat P, Vivante A, Hilger AC (2014). Mutations in 12 known dominant disease-causing genes clarify many congenital anomalies of the kidney and urinary tract. Kidney Int.

[10] Van der Ven AT, Connaughton DM, Ityel H, Mann N, Nakayama M, Chen J (2018). Whole-exome sequencing identifies causative mutations in families with congenital anomalies of the kidney and urinary tract. J Am Soc Nephrol.

[11] Zhou X, Wang Y, Shao B, Wang C, Hu P, Qiao F (2020). Molecular diagnostic in fetuses with isolated congenital anomalies of the kidney and urinary tract by whole-exome sequencing. J Clin Lab Anal.

[12] Lei TY, Fu F, Li R, Yu QX, Du K, Zhang WW (2020). Whole-exome sequencing in the evaluation of fetal congenital anomalies of the kidney and urinary tract detected by ultrasonography. Prenat Diagn.

